# Strain differences in the collective behaviour of zebrafish (*Danio rerio*) in heterogeneous environment

**DOI:** 10.1098/rsos.160451

**Published:** 2016-10-12

**Authors:** Axel Séguret, Bertrand Collignon, José Halloy

**Affiliations:** University Paris Diderot, Sorbonne Paris Cité, LIED, UMR 8236, 75013, Paris, France

**Keywords:** collective behaviour, decision-making, zebrafish, phenotypes

## Abstract

Recent studies show differences in individual motion and shoaling tendency between strains of the same species. Here, we analyse collective motion and response to visual stimuli in two morphologically different strains (TL and AB) of zebrafish. For both strains, we observed 10 groups of 5 and 10 zebrafish swimming freely in a large experimental tank with two identical landmarks (cylinders or discs) for 1 h. We tracked the positions of the fish by an automated tracking method and compute several metrics at the group level. First, the probability of the presence shows that both strains avoid free space and are more likely to swim in the vicinity of the walls of the tank and the landmarks. Second, the analysis of landmarks occupancy shows that AB zebrafish are more present in their vicinity than TL ones and that both strains regularly transit from one to the other one with no preference on the long duration. Finally, TL zebrafish show a higher cohesion than AB zebrafish. Thus, environmental heterogeneity and duration of the trials allow to reveal individual and collective behavioural variabilities among different strains of zebrafish. These results provide a new insight into the need to take into account individual variability of zebrafish strains for studying collective behaviour.

## 1. Introduction

Collective decision-making has been evidenced in many animal species and contexts [1] including food collection [2], problem-solving [3,4], collective movement [5–11] or nest-site selection [12,13]. In this later case, social animals have to select a resting site among several potential options in a complex environment. This selection can be made either through individual decisions or complex decision-making processes involving the participation of all individuals [14] and can be temporary or permanent according to the needs and living style of the considered species.

This process of collective decision has been studied for a long time in social animals that select a permanent home: social insects, fish and birds. In particular, experiments on fish have been generally designed to observe preferences for particular environmental features or landmarks for a relatively short experimental time (few seconds [15], 5 min [16], 10 min [17], up to 30 min per trial [18]). These studies have shown, for example, that landmarks in a bare tank arouse interest and attract the fish [18,19] and that variations of the shape of these landmarks can change territory features [20,21].

While these studies provide numerous insights on the individual and collective preferences in fish groups, they generally rely on the comparison between two or more qualitatively different alternatives. Thus, the selection of one option is often based on an intrinsic preference for a particular feature in comparison with the others. Such asymmetric choices may hide the collective decision that results from the internal processes of decision-making of the group.

Furthermore, it has been evidenced with fish (*Oreochromis niloticus*, *Gambusia holbrooki* and *Notemigonus crysoleucas*) that the group sizes impact motion [22,23] (speed, turning speed and nearest-neighbour distance) and some moving behaviours [24], such as the milling and the alignment. Depending on the species, studies show opposite results: Becco *et al*. [22] work with *Oreochromis niloticus* with two group sizes (330 and 905 fish); Tunstrom *et al*. [24] work with *Notemigonus crysoleucas* with four group sizes (30, 70, 150 and 300 fish). Increasing the group size of *Oreochromis niloticus* makes stronger alignments, while for *Notemigonus crysoleucas* alignments decrease. In this study, we test whether and how collective behaviours and decision-making are affected by the strain and the group sizes.

Here, our aim is to characterize the collective behaviour of groups of zebrafish swimming in an environment with identical landmarks. We observe the collective motion of small shoals of different group sizes (5 and 10 fish) and of two different zebrafish strains (laboratory wild-type AB or TL). Zebrafish are a gregarious vertebrate model organism that can be used to study the cohesion of the group and its decision-making [25,26]. Originating from India, the zebrafish is a diurnal species that prefers staying in groups both in nature and in the laboratory [9,27,28]. There is a wide variability in shoal sizes: zebrafish live in small groups in shallow freshwaters [29,30] and can aggregate into larger groups of 300 individuals [31]. Zebrafish living in a variety of habitats with varying structural complexities [31,32] (from river channels, irrigation canals to beels), we set our experimental method relying on the observations of fish freely swimming in an open environment along with heterogeneous landmarks rather than in a constraining set-up (i.e. mazes as used in [15–17]). We observe for 1 h per replicate each group of fish swimming in a large experimental tank (1×1 m and 1.20×1.20 m) with two spots of interest (landmarks).

The landmarks consist of two striped yellow-green opaque plastic cylinders placed in the water column or two blue transparent floating Perspex discs providing shadow. We choose these colours in the visible spectrum of the zebrafish according to the results of [33]. We expect that these landmarks placed in a homogeneous environment could induce a choice of one preferred option by the zebrafish as evidenced for other species faced with identical resources [13,34,35]. On the one hand, we test with cylinders the effect of visual and physical cues in the water column on collective choices. Since zebrafish are known to swim along the walls of the experimental tank [36], cylinders could act as such walls in the water column. On the other hand, we test with floating discs the impact of visual and physical cues above water, on collective choices. We placed discs and cylinders as landmarks to see whether and how the two strains of zebrafish will adapt their group behaviour and their preferences for landmarks.

## 2. Results

### 2.1. Differences of group features between AB and TL strains of zebrafish

**Figure 1.**
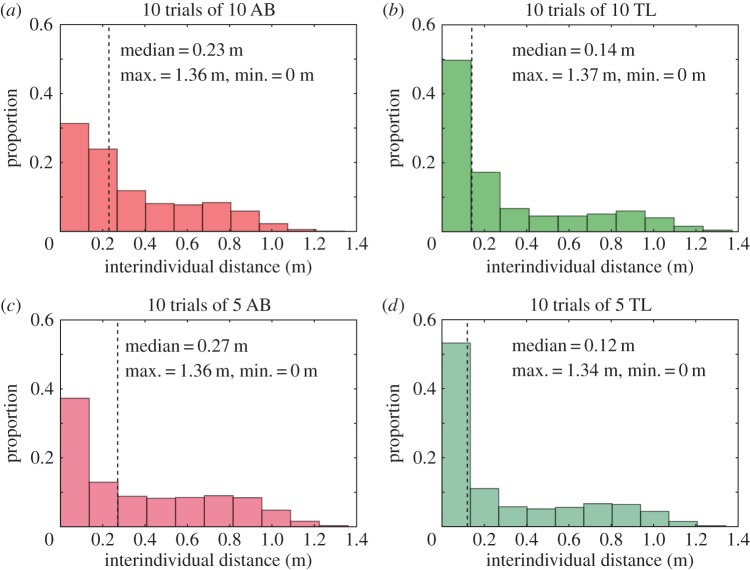
Interindividual distances for 10 trials in the square arena with two cylinders with (*a*) 10 AB zebrafish (*N*=22 480 363 distances), (*b*) 10 TL zebrafish (*N*=20 981 798 distances), (*c*) 5 AB zebrafish (*N*=5 169 077 distances) and (*d*) 5 TL zebrafish (*N*= 4 578 036 distances). Where *N* is the number of measured distances. The distributions show that groups of TL zebrafish have a stronger cohesion than groups of AB zebrafish. Although group size does not change this distribution in groups of TL strain, it has a strong impact on the AB strain. Distributions of the interindividual distances of 10 AB and 10 TL are different, 5 AB and 5 TL are different, 5 AB and 10 AB are different and 5 TL and 10 TL are different (see in the text the results of the statistical tests).

We test two types of landmarks: striped yellow and green cylinders and floating Perspex transparent blue discs (see Material and methods), on two laboratory strains of zebrafish (TL and AB) for 10 replicates. We computed the interindividual distances between all fish to characterize the cohesion of the group for both strains and group sizes in the presence of the cylinders. The distribution of all interindividual distances (figure 1) shows that the groups of TL zebrafish have a stronger cohesion (5 TL: median=0.12 m and 10 TL: median=0.14 m) than the groups of AB zebrafish (5 AB: median=0.27 m and 10 AB: median=0.23 m). The intrastrain comparison for the two group sizes shows that the distributions significantly differ from each other (Kolmogorov–Smirnov test, 5 AB versus 10 AB, *D*=0.102, *p*<0.001; 5 TL versus 10 TL, *D*=0.080, *p*<0.001). The interstrain comparison for similar group sizes also reveals a statistical difference between the distributions (Kolmogorov–Smirnov test, 10 AB versus 10 TL, *D*=0.184, *p*<0.001; 5 AB versus 5 TL, *D*=0.161, *p*<0.001).

**Figure 2.**
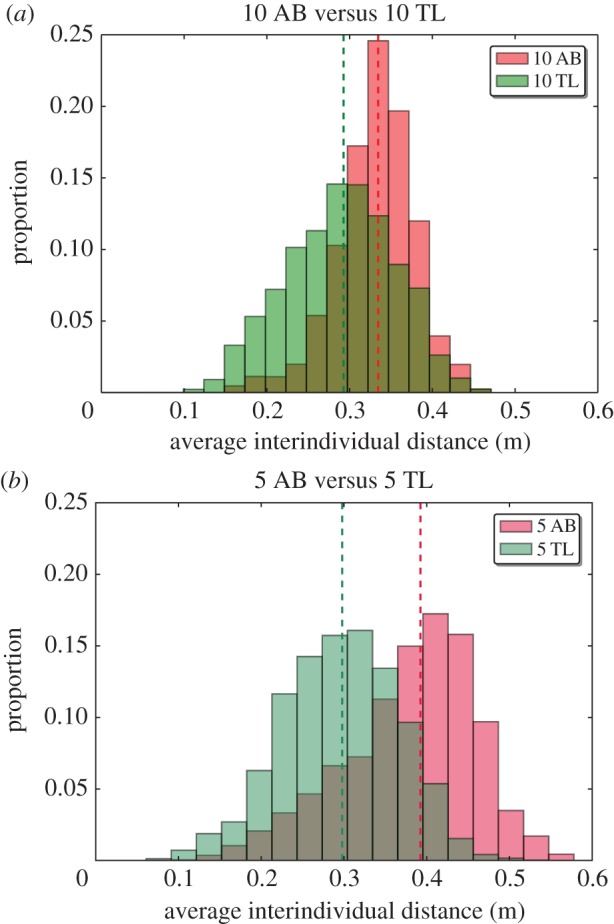
Average interindividual distance in the square arena with two cylinders (*a*) for 10 AB zebrafish (*N*=518 960 measurements) versus 10 TL zebrafish (*N*=500 794 measurements). The red distribution represents 10 trials with groups of 10 AB zebrafish and the green represents 10 trials with groups of 10 TL zebrafish. (*b*) For 5 AB zebrafish (*N*=528 357 measurements) versus 5 TL zebrafish (*N*=495 659 measurements). The red distribution represents 10 trials with groups of 5 AB zebrafish and the green is for 10 trials with groups of 5 TL zebrafish. Dashed lines represent medians. TL zebrafish are more cohesive than AB zebrafish. Smaller groups of AB zebrafish show a shift to higher values. The distributions of the average interindividual distances of 10 AB and 10 TL are different, 5 AB and 5 TL are different, 5 AB and 10 AB are different and 5 TL and 10 TL are different.

The distributions of the average interindividual distances measured at each time step confirm these results (figure 2, Kolmogorov–Smirnov test, 5 AB versus 10 AB, *D*=0.433, *p*<0.001; 5 TL versus 10 TL, *D*=0.051, *p*<0.001; 10 AB versus 10 TL, *D*=0.333, *p*<0.001; 5 AB versus 5 TL, *D*=0.464, *p*<0.001). The medians of the distributions of the average interindividual distances for 10 AB is 0.33 m, for 10 TL is 0.29 m, for 5 AB is 0.39 m and for 5 TL is 0.30 m. Moreover, the time series of the average interindividual distance reveals that the cohesion of the fish decreases for both strains and both group sizes through the time (electronic supplementary materials, figure S11).

**Figure 3.**
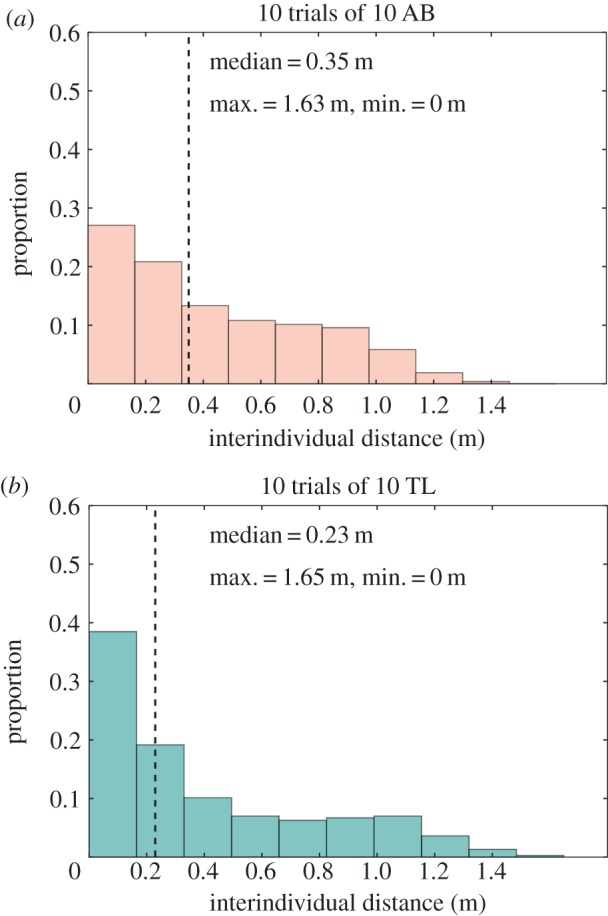
Interindividual distances for 10 trials in the square arena with two discs with groups of (*a*) 10 AB zebrafish (*N*=1 620 450 distances), (*b*) 10 TL zebrafish (*N*=1 620 450 distances) in a tank with two discs. *N* is the number of measurements of distance. Distribution of the interindividual distance shows that groups of TL zebrafish have a stronger cohesion than groups of AB zebrafish. Max. and min. show the maximum or minimum distances found between fish, respectively. A Kolmogorov–Smirnov test shows that the distributions of the interindividual distances of 10 AB and 10 TL are different.

**Figure 4.**
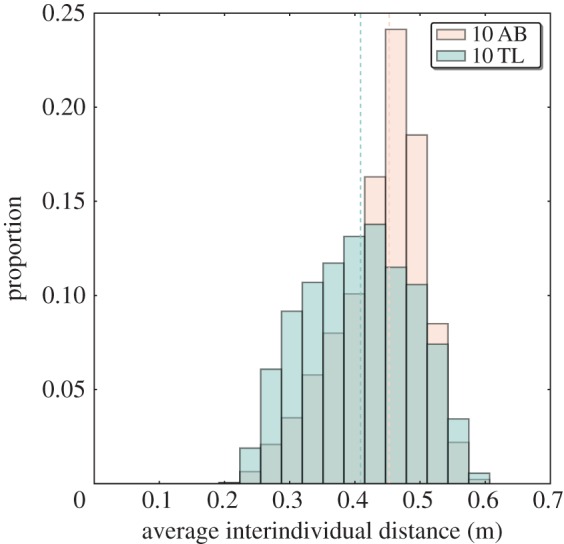
Average interindividual distance in the square arena with two discs for 10 AB zebrafish (*N*=36 010 measurements) versus 10 TL zebrafish (*N*=36 010 measurements). The orange distribution represents 10 trials with groups of 10 AB zebrafish and the blue-green represents 10 trials with groups of 10 TL zebrafish. Dashed lines represent medians. TL zebrafish are more cohesive than AB zebrafish. Smaller groups of AB zebrafish show a shift to higher values. The distributions of the average interindividual distances of 10 AB and 10 TL are different.

In the presence of the floating discs, TL zebrafish were also significantly more cohesive than the AB zebrafish, as shown by the distribution of the interindividual distances (medians for 10 AB: 0.35 m, for 10 TL: 0.23 m, figure 3, Kolmogorov–Smirnov test, *D*=0.135 and *p*<0.001). The previous analysis matches with the one obtained for the measurements of average interindividual distances (medians for 10 AB: 0.45 m, for 10 TL: 0.41 m, figure 4, Kolmogorov–Smirnov test, *D*=0.230 and *p*<0.001).

### 2.2. Group sizes affect strain preferences for landmarks

**Figure 5.**
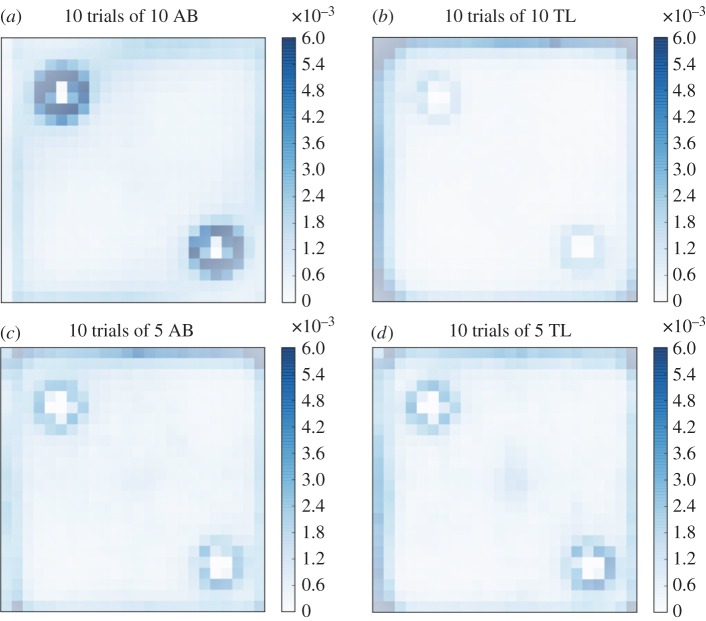
Probability of the presence of (*a*) 10 AB zebrafish, (*b*) 10 TL zebrafish, (*c*) 5 AB zebrafish and (*d*) 5 TL zebrafish in a tank with two cylinders. The probability is calculated on the positions of all zebrafish (i.e. 5 or 10 individuals) observed for 1 h and cumulated for 10 trials. The response to the landmarks is strain and group size dependent: while 5 AB and 5 TL zebrafish show similar probability of the presence near the cylinders, a larger group size of AB increases the response to the landmarks but decreases the response of groups of 10 TL.

**Figure 6.**
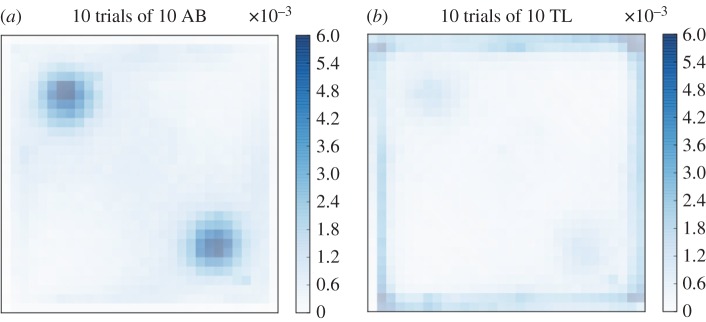
Probability of the presence of (*a*) 10 AB zebrafish in a tank with two discs, (*b*) 10 TL zebrafish in a tank with two discs. The probability is calculated on the positions of all zebrafish observed for 1 h and cumulated for 10 trials. Attractivity to landmarks is strain dependent: the probability of the presence of finding 10 AB zebrafish around the landmarks is two times higher than that of 10 TL zebrafish.

In the presence of the two cylinders, the groups of 10 AB zebrafish are mainly present along the wall of the tank and around the cylinders, as shown by their average probability of the presence computed for the 10 replicates, each of 1 h observation time (figure 5). On the contrary, groups of 5 TL, 10 TL and 5 AB zebrafish are less observed near the cylinders but are still present along the walls of the tank (figure 5). With the two discs we observed similar behaviours than in the previous experiments with cylinders. The maximum probability of the presence under the discs with AB zebrafish reaches 4.5×10^−3^ while for TL zebrafish it reaches only 1×10^−3^ (figure 6). Again, TL zebrafish spent the majority of their time near the borders of the tank. The probabilities of the presence computed for each experiment are shown in the electronic supplementary materials, figures S1–S6.

**Figure 7.**
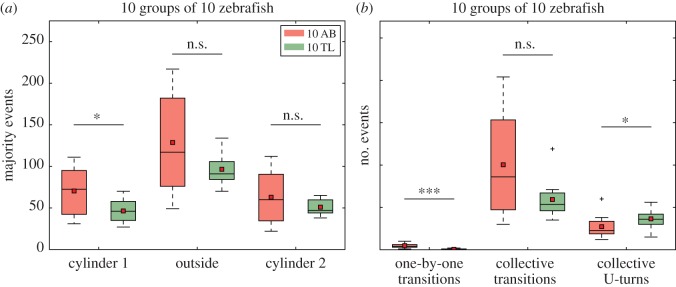
Landmark occupancy and transitions for 10 trials of 10 AB zebrafish and 10 TL zebrafish. (*a*) Number of majority events occurring around the cylinders and outside. A majority event was considered as soon as more than or equal to 7 fish are aggregated in the same zone. (*b*) Number of transitions of the majority from one zone to another one. We mainly looked at ‘*One-by-one*’ *transitions* (the fish transit one by one from one cylinder to the other), *Collective transitions* (the whole group transits between both cylinders through the outside area) and *Collective *U*-turns transitions* (the group go back to the previous cylinder). For both strains, while several U-turns were observed, the majority of transitions were made in groups (Collective transitions) and only a few were made one by one. The TL strain significantly differs from the AB strain by performing significantly less One-by-one transitions and more Collective U-turns. Each boxplot is composed of 10 values of majority events or numbers of events. *p<0.05, **p<0.01, ***p<0.001, n.s., non-significant.

Then, we studied in more detail the dynamics of the presence of groups of 10 zebrafish near the cylinders. The fish of the AB strain form a cohesive group that regularly transits from one landmark to the other one at the beginning of the trial. Then the group starts to split into multiple subgroups and the periodicity of the visits becomes less regular (electronic supplementary material, figure S7). These oscillations are also observed for groups of the TL strain but contrarily to AB zebrafish, this phenomenon is observed for the whole experimental time (electronic supplementary material, figure S8). To quantify the dynamics of these transitions, we computed the number of majority events detected near one of the two landmarks or outside of them. A majority event was counted when 7 or more individuals were simultaneously present in the same zone independently of the duration of this majority event. Figure 7*a* shows that the median and mean number of majority events are always smaller with the TL strain, but this difference is only significant for the majorities detected near one of the cylinders (cylinder 1, Mann–Whitney *U*-test, *U*=26, *p*=0.038 ; cylinder 2, Mann–Whitney *U* test, *U*=48, *p*=0.455 ; outside, Mann–Whitney *U*-test, *U*=38, *p*=0.192). We also characterized the transitions of the fish from one landmark to the other one by analysing the succession of majority events. In particular, we counted the number of ‘Collective transitions’ (i.e. two majority events nearby different cylinders separated by a majority outside), the number of ‘One-by-one transitions’ (i.e. succession of a majority event in one cylinder and a majority event in the other cylinder) and finally the number of ‘Collective U-turns’ (i.e. two majority events in the same cylinder separated by a majority outside). It reveals that the main transitions occurring for AB zebrafish are the collective ones while some collective U-turns and almost no individual transitions were detected (figure 7*b*, red). Similarly, almost no individual transition was observed for the TL zebrafish that perform mainly collective transitions (figure 7*b*, green). TL zebrafish also performed numerous collective U-turns. The absence of individual transitions reveals that both strains are mostly swimming in groups but with different collective dynamics. We compared the results with Mann–Whitney *U*-tests: ‘One-by-one transitions’ (*U*=3.0, *p*<0.001) and ‘Collective U-turns’ (*U*=27.5, *p*<0.050) between AB and TL are significantly different while ‘Collective transitions’ between AB and TL are not (*U*=40, *p*=0.236).

**Figure 8.**
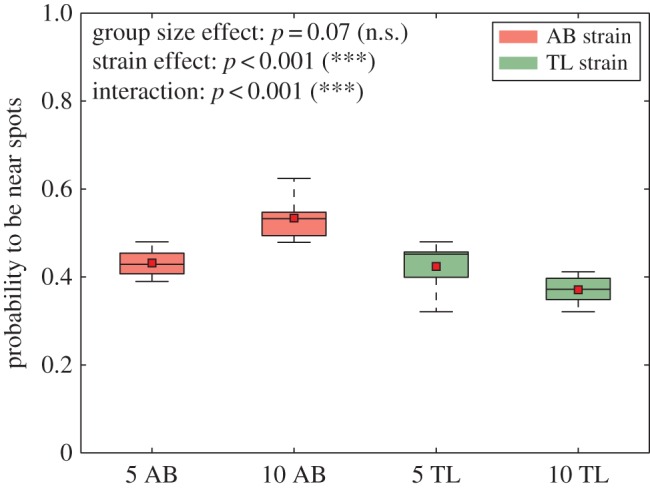
Probability to be at 25 cm from the centre of the cylinders for 10 trials with 5 AB, 10 AB, 5 TL or 10 TL zebrafish in a 1 m^2^ tank with two cylinders. The black line shows the median, the red square shows the mean. Statistical tests show that groups of 10 AB are more attracted by the cylinders than groups of 5 AB and 5 TL which are more attracted by cylinders than groups of 10 TL. *N*=10 measurements for 5 AB, 10 AB, 5 TL and 10 TL. Each boxplot is composed of 10 values of probability. *p<0.05, **p<0.01, ***p<0.001, n.s., non-significant.

To highlight the differential effects of the strains and the group sizes on space fidelity around cylinders, we measured the proportion of positions that were detected at 25 cm from the centre of the cylinders (figure 8). This measurement confirms that groups of 10 AB zebrafish were more present near the cylinders than groups of 5 AB. By contrast, while groups of 5 TL responded similarly to groups of 5 AB, groups of 10 TL zebrafish were less detected near the cylinders. A two-way ANOVA (group size, strain, *n*=10 for each experimental condition) indicated a non-significative effect of the group size (*p*=0.07, *F*=3.59, d.f.=1) but a significant effect of the strain (*p*<0.001, *F*=43.17, d.f.=1) and a significant strain/group size interaction (*p*<0.001, *F*=35.38, d.f.=1) on the attractivity of the cylinders. The interaction effect indicates here a strain-specific effect of the group size on the time spent near the landmarks: groups of 10 AB are more attracted by the cylinders than groups of 5 AB, but on the contrary, 10 TL are less detected near the cylinders than groups of 5 TL. To confirm this observation, we compared the proportions for all groups (5 or 10 zebrafish, AB or TL strain) with a one-way ANOVA. The test confirmed that the size of the group and the type of strain have a significant influence on the attractivity of the cylinders (*p*<0.001, *F*=27.38, d.f.=3). Finally, a Tukey’s honest significant difference criterion *post hoc* test shows that attractivity of the cylinders is always different except for the couple 5 AB versus 5 TL. This series of tests confirmed that groups of 10 AB are more attracted by the cylinders than groups of 5 AB and 5 TL which are more attracted by cylinders than groups of 10 TL.

**Figure 9.**
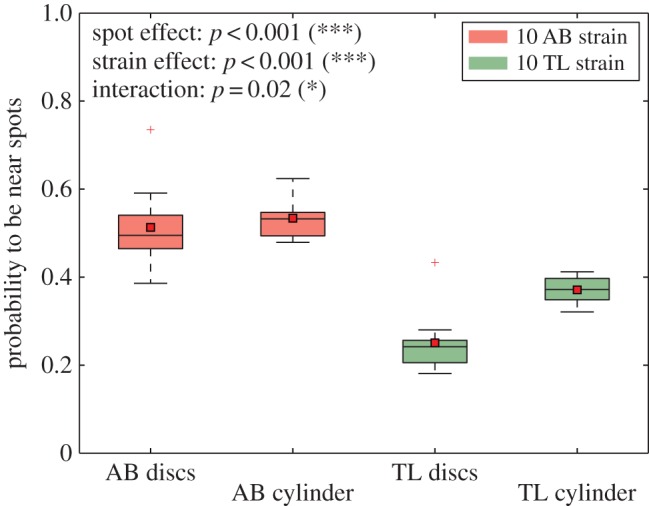
Probability to be at 25 cm from the centre of cylinders or discs for 10 trials. Groups of 10 AB or 10 TL zebrafish in a tank with two cylinders or two discs. The black line shows the median, the red square shows the mean. A series of tests shows that groups of 10 AB are attracted by cylinders as much as discs, groups of 10 TL are more attracted by cylinders than discs, groups of 10 AB are more attracted by the cylinders and the discs than groups of 10 TL. *N*=10 measurements for AB disc, AB cylinder, TL disc and TL cylinder. Each boxplot is composed of 10 values of probability *p<0.05, **p<0.01, ***p<0.001, n.s., non-significant.

Finally, we compared the probability to be near the landmarks (cylinders or discs) for 10 AB and 10 TL zebrafish with a two-way ANOVA (figure 9). It revealed that the type of landmarks (*p*<0.001, *F*=11.37, d.f.=1) and the strain of zebrafish affect the attraction (*p*<0.001, *F*=102.95, d.f.=1), while there is also evidence of an interaction effect between type of landmarks and strains (*p*=0.02, *F*=5.67, d.f.=1). To confirm this observation, we compared the proportions for all groups (10 zebrafish, AB or TL strain in the presence of cylinders or discs) with a one-way ANOVA. The test confirmed that the type of the strain and the type of the landmark have a significant influence on the attractivity of the cylinders (*p*<0.001, *F*=39.99, d.f.=3). Finally, a Tukey’s honest significant difference criterion *post hoc* test shows that all paired comparisons are significantly different except for the 10 AB discs and 10 AB cylinders. This series of tests confirmed that groups of 10 AB zebrafish are more attracted by the landmarks than groups of 10 TL which show more attraction for cylinder type than disc type.

## 3. Discussion

We investigated whether collective motion and collective choice can differ in groups of 5 and 10 individuals of the same species (zebrafish *Danio rerio*) but of different strains (AB versus TL) in the presence of two identical landmarks. One hour observation trials show that the zebrafish groups do not select one of the two landmarks. The fish were mainly swimming together and oscillating from one landmark to the other with short resting times. Thus, while all individuals can be present at the same landmark for a few seconds (electronic supplementary material, S7 and S8), the probability of the presence computed for the entire experimental time shows that the fish were equally present at both stimuli. Therefore, no collective choice emerged on the long duration for both strains of zebrafish and group sizes. Hence, long or short study durations can reveal opposite conclusions on collective motions. In other species, site fidelity has been studied for longer time period. It has been reported that *Hemigrapsus sanguineus* exhibited low site fidelity after 24 h: 17.8% and 2.3% of the released crabs were recovered within 0.1 m from the two studied sites [37]; for *Helograpsus haswellianus*, the authors have shown that the recapture rates of marked crabs were very low across their four week lunar study (5.8% and 5.3% on two different sites at a distance of 70 km), while recaptures often occurred in the same trap (31.3 and 25%) [38]. The authors suggest that this low rate of site fidelity could be due to foraging issues. For *Genyonemus lineatus*, researchers report evidence of site fidelity during the first 7 days of their study (over 240 days) with low and variable residency times [39]. For *Cynoscion regalis*, it has been found that there is a strong link between the birth location of the parents and their spawning location: from 60 to 81% [40].

Our methodology is complementary to typical Y-maze experiments. We extend and compare their conclusions to our observations with repeated interactions between the fish and their environment. During an hour, the collective behaviour of zebrafish contrasts with other collective species in which spatial fidelity emerges from the interactions between the individuals that take place in the resting sites (in cockroaches [41], in hymenoptera [12]). These oscillations from one site to the other one could originate from individual differences among group members: *bold* and *shy* behavioural profiles have been evidenced in zebrafish according to the intrinsic propensity of each fish to explore new environments [42,43]. It has been shown that zebrafish with high boldness lost their shoal cohesion when making decisions, unlike other species (*Gasterosteus aculeatus*) where boldness as attractor has been identified [44]. In this context, the presence of bolder fish in the group could favour the transition from one spot to the other one while groups composed only by shy individuals could show less frequent departures [45]. Boldness and shyness as modifiers of group behaviour occur also in mammalians, for example, where bold sheep tend to split big groups into smaller subgroups of equal size [46]. We did not identify significant differences between both strains for collective transitions but we revealed significant differences for the ‘one-by-one’ transitions and the collective U-turns. AB zebrafish show more ‘one-by-one’ transitions than TL and TL zebrafrish show more collective U-turns than AB. We could categorize the ‘one-by-one’ transitions as more dangerous because they occur when the group transit from a landmark to the other one, one fish after another. The fish are thus exposed in the tank and we could link these transitions with bold personalities. Following the same reasoning, collective U-turns can be assimilated as shy events because fish go back to their previous known location. Hence, there could be a difference of boldness between both strains that might be highlighted by a deeper analysis of the transitions.

A more detailed analysis shows quantitative differences among the two studied strains and group sizes. Concerning the response of the fish to the landmarks, we highlight that groups of 5 AB and TL zebrafish show the same attraction to the cylinders by computing the probability for the fish to be observed near these landmarks. This attraction increases for groups of 10 AB zebrafish but decreases for groups of 10 TL zebrafish. This strain difference is also observed in the experiments with floating discs. In addition, the type of the landmarks seems to be determinant for TL zebrafish as they prefer objects immersed in the water column to objects lying on the surface of the water. Hence, differences of collective behaviour between the two tested strains of zebrafish exist.

These different responses to heterogeneities may be based on the intrinsic preference of the fish of a particular strain for congeners or for landmarks. Such difference has already been shown in shoaling tendency between several strains of guppies [47] and zebrafish [48,49]. In their natural environment, fish have to balance the costs of risks and benefits of moving in groups or staying near landmarks [50]. Our results suggest that there are more collective transitions than collective U-turns and ‘one-by-one’ transitions. It means that zebrafish (whatever the strain) prefer to transit in a group and thus avoid group fission when crossing an open area. Moving in groups first of all prevents the fish from being static prey, and second allow spatial recognition and easier food and predator detection. The drawback is that the takeover of the fish on the territory is punctual and they have less chance to find areas where they can hide. Staying around landmarks gives the fish a feeling of control of the territory and the possibility to hide from predators. In that case the drawback is that the prey will rarely cross the territory of the fish.

Regarding the structure of the group, we notice that whatever the group size of TL zebrafish, the cohesion of the group does not change and is always stronger than those of groups of AB zebrafish. Also, the bigger the group of AB zebrafish, the stronger the cohesion. These differences of group cohesion may be based on differences of physical features between AB and TL zebrafish. Cohesion differences could be explained by phenotype differences between the two strains. Some studies demonstrated that a large variability exists in the individual motion and shoaling tendency of the zebrafish according to their age or strain. For example, adults AB and casper zebrafish swim longer distance than ABstrg, EK, TU or WIK zebrafish [51]. Likewise, the interindividual distance between shoal members decreases from 16 body length to 3.5 body length between day 7 and five months after fertilization [52]. Also, it has been demonstrated that the fin size has an impact on the swimming performance and the behaviour of the zebrafish [53]. AB and TL zebrafish show different fin lengths and different patterns on the skin: TL zebrafish are homozygous for leo^t1^ and lof^dt1^, where leo^t1^ is a recessive mutation causing spotting in adult zebrafish and lof^dt1^ is a dominant homozygous viable mutation causing long fins [54,55]. Thus, AB zebrafish have short fins and TL zebrafish show long fins. One may suggest that TL zebrafish move a higher quantity of water with their long fins when swimming and thus emit a stronger signal of presence (hydrodynamical signal). Thus, it may be easier for conspecifics in the moving shoal to perceive the signal through their lateral line and realign themselves according to their conspecifics. If the realignment becomes easier, it is simpler for TL zebrafish to keep their position in the shoal, which increases its cohesion. Following a similar hypothesis, the signal of presence is weaker for AB zebrafish due to their shorter fins. Thus, realignment in the moving shoal is less performant and their cohesion decreases. Hemmings [56] and Partridge & Pitcher [57] showed that fish use vision for attraction and the lateral line for repulsion. Recently, it has been revealed that the visual field (binocular and lateral) modify the group cohesion [58]. The zebrafish strain showing the bigger visual field should be less cohesive. However, none of the previous mutations (leo and lof) could explain these differences of visual fields in both strains of zebrafish. The lateral line could also explain the differences of cohesion between AB and TL zebrafish: the mutation of the leo gene (TL zebrafish) may have an effect on it, lowering its performance. Hence, unlike the AB zebrafish, the repulsion may occur when TL zebrafish are closer to each other, forcing them to be more cohesive. Each of these hypotheses could explain the collective behaviours observed during the experiments and nothing prevents merging all of them.

Finally, we did not quantify aggressive behaviour during the experiments. There are several studies trying to find a link between aggression and shoal size. Morgan [59] shows that aggressive interactions of *Pimephales notatus* decrease significantly as shoal size increases, while Rehnberg & Smith [60] show that there is no clear evidence between levels of aggression and shoal size with *Danio rerio*. The issue of aggressive behaviour could be left open for another study.

In conclusion, this study demonstrates that behavioural differences exist at the individual and collective levels in the same species of animal. The analysis of the dynamics reveals that AB and TL zebrafish mainly oscillate in groups between landmarks. In addition, increasing the size of the group leads to opposite results for the two strains: groups of 10 AB zebrafish are proportionally more detected near the landmarks than groups of 5 AB while groups of 10 TL zebrafish are less attracted by the landmarks than groups of 5 TL. Finally, the two tested zebrafish strains show differences at the structural level: (1) groups of TL zebrafish are more cohesive than groups of AB zebrafish and (2) AB zebrafish collective responses to landmarks show that they are generally more present near the cylinders and floating discs than TL zebrafish. Thus, this study provides evidence that zebrafish do not select resting site on the mid-term and highlights behavioural differences at the individual and collective levels among the two tested strains of zebrafish. Future studies of collective behaviour should consider the tested strains, the intra-strain composition of the shoals and the duration of each trial.

## 4. Material and methods

### 4.1. Fish and housing

We acquired 500 adult common laboratory wild-type zebrafish (200 AB strain and 300 TL strain) from Institut Curie (Paris) and raised them under the same conditions in tanks of 60 l by groups of 50. The zebrafish AB line show a zebra skin, short tail and fin. The zebrafish TL line show a spotted skin, long tail and fin and barbel. Both strains are 3.5 cm long. The zebrafish used for the experiments are adult fish between 5 and 18 months of age. During this period, zebrafish show a shoaling tendency allowing study of their collective behaviours. We kept fish under laboratory condition, 27°C, 500 μS salinity with a 9 D : 15 L cycle. The fish were fed two times per day (Special Diets Services SDS-400 Scientific Fish Food). Water pH is maintained at 7.5 and nitrites (NO^2−^) are below 0.3 mg l^−1^. We measured the size of the caudal fins of 10 AB (about 0.4 cm) and 10 TL (about 1.1 cm) zebrafish.

### 4.2. Experimental set-up

The experimental tank consists of a 1.2×1.2 m tank confined in a 2×2×2.35 m experimental area surrounded by a white sheet, in order to isolate the experiments and homogenize luminosity. The wall of the experimental tank was covered with white tape and the water column is 6 cm. Water pH is maintained at 7.5 and nitrites (NO^2−^) are below 0.3 mg l^−1^. The experiments with discs were performed in the experimental tank, while those with cylinders were performed in a white square arena (1×1×0.15 m) placed in the experimental tank. Groups of zebrafish were randomly formed at the beginning of the experiments.

The experiments were recorded by a high-resolution camera (2048×2048 px, Basler Scout acA2040-25gm) placed above the experimental tank and recording at 15 fps (frame per second). Luminosity is ensured by 4 fluorescents lamps of 80 W placed on each side of the tank, on the floor and directed towards the walls to provide indirect lighting.

**Figure 10.**
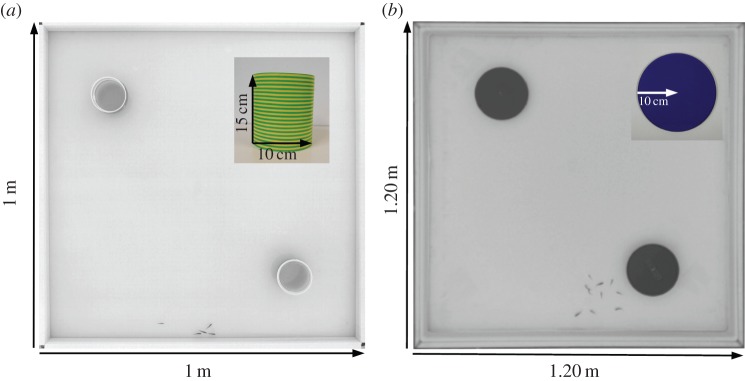
(*a*) Experimental set-up (1×1 m) with two cylinders symmetrically placed and a magnified cylinder (*ϕ*=10 cm, height= 15 cm). (*b*) Experimental set-up (1.2×1.2 m) with two blue Perspex discs symmetrically placed and a disc (*ϕ*=20 cm). In the experimental tank, the water column is 6 cm. Luminosity is ensured by 4 fluorescents lamps of 80 W placed on each side of the tank, on the floor and directed towards the walls to provide indirect lighting. The whole set-up is confined in a 2×2×2.35 m experimental chamber (cage) surrounded by white sheets to isolate the experiments and to homogenize luminosity.

To trigger interest of fish, we placed symmetrically in the set-up either two floating discs (*ϕ*=20 cm) or two cylinders (*ϕ*=10 cm, height=15 cm) surrounded by yellow- and green-striped tape [61–63]. To avoid the presence of a blind zone, the cylinders were slightly tilted towards the centre of the tank. The centre of both discs and cylinders are at $25\sqrt{2}\;\mathrm{cm}$ from two opposite corners along the diagonal of the tank (figure 10).

### 4.3. Experimental procedure

We recorded the behaviour of zebrafish swimming in the experimental tank for 1 h. Before the trials, the attractive landmarks are put in the set-up and fish are placed with a hand net in a cylindrical arena (20 cm diameter) made of Plexiglas placed in the centre of our tank. Following a 5 min acclimatization period, this arena is removed and the fish are able to freely swim in the experimental arena. We performed 10 trials for each strain with the floating discs and 10 trials for each combination of parameters (number of fish×strain) with the cylinders for a total of 60 experiments. Each fish was never tested twice in the same experimental condition.

### 4.4. Tracking and data analysis

The methodology based on massive data gathering [64] has now become standard in studies on animal collective behaviour with flies, *Drosophila melanogaster* [65,66], birds, *Sturnus vulgaris* [67–69] and fish, *Notemigonus crysoleucas* [70]. The experiments with cylinders were recorded at 15 fps and tracked online by a custom-made tracking system based on blob detection. We call a batch a group of 10 experiments. For these batchs, each experiment consists of 540 000 positions (10 zebrafish×54 000 frames) and 270 000 positions (5 zebrafish×54 000 frames). For experiments with discs, we faced tracking troubles. Since the fish below the floating discs were difficult to distinguish by the program due to a lack of sufficient contrast, experiments with floating disc were tracked offline by two custom Matlab scripts. A first script automatically identifies the positions of the fish swimming outside of the floating discs by blob detection. Since this method did not allow a perfect detection of all the individuals, we developed a second script that was run after the first one and that plotted the frame where a fish (or more) was undetected for the user to manually identify the missing individual(s). It allowed us to identify the fish that were partially hidden during a collision/superposition with another fish or the fish that were situated under the floating discs. Since this analysis tool is time-costly, we only analysed 1 fps for all experiments with discs. For these batchs, each experiment consists of 36 000 positions (10 zebrafish×3600 positions).

Since our tracking system did not solve collision with accuracy, we did not calculate individual measurements but characterized the aggregation level of the group. The probability of the presence of the fish was calculated by the cumulated positions of all individuals along the entire experiment. We also calculated the distance between each individual fish and the attractive landmarks (and averaged it) as well as the interindividual distances between the fish and the average interindividual distance. Finally, we computed the time of shelter occupancy as the time that is spent by the fish at less than 25 cm from the attractive landmarks. These time sequences were calculated according to the number of fish present near the landmarks. All scripts were coded in Python using scientific and statistic libraries (numpy, pylab, scilab and matplotlib).

To compute the number of majority events, the number of fish was averaged over the 15 frames of every second. This operation guarantees that a majority event is ended by the departure of a fish and not by an error of detection during one frame by the tracking system. Electronic supplementary material, figures S9 and S10 show the proportions of the durations of the majority events before and after this interpolation.

### 4.5. Statistics

For figures 8 and 9, ten measurements of means of the probability for different groups of zebrafish to be near the landmarks are plotted. They have been tested using a two-way ANOVA. We then compared the data between each group using a one-way ANOVA and finally used a Tukey’s honest significant difference criterion *post hoc* test. We did these tests on Matlab and chose 0.001 as significance level. In the electronic supplementary material, table S1, we show the number of majority events after interpolation of the data at 1 fps. This table is related to figure 7. We used Mann–Whitney *U* tests to compare the number of events between strains, areas and transition types. These tests are performed on 10 values of majority events for each strain, area and transition type. These tests were made with the Python package scipy. We chose 0.001 (***), 0.01 (**) and 0.05 (*) as significance levels. For figure 1 as well as figures 2 and 3, we compared the distribution with Kolmogorov–Smirnov tests. These tests were made with the Python package scipy. We chose 0.001 as significance level.

## Supplementary Material

one file: Supplementary figures of “Strain differences in the collective behaviour of zebrafish (Danio rerio) in heterogeneous environment”.

## Data accessibility

Supporting data will be available from the Dryad Digital Repository: http://dx.doi.org/10.5061/dryad.01j61 [71].
