# Supplementary material for: Strain differences in the collective behaviour of zebrafish (Danio rerio) in heterogeneous environment
Source: R Soc Open Sci. 2016 Oct 12;3(10):160451. doi: 10.1098/rsos.160451 (PMC5098983; doi:10.1098/rsos.160451)
Supplement: one file: Supplementary figures of “Strain differences in the collective behaviour of zebrafish (Danio rerio) in heterogeneous environment”. [file rsos160451supp1.pdf]

Supplementary material

Supplementary figures of "Strains differences in the collective behaviour of zebrafish (*Danio rerio*) in heterogeneous environment".

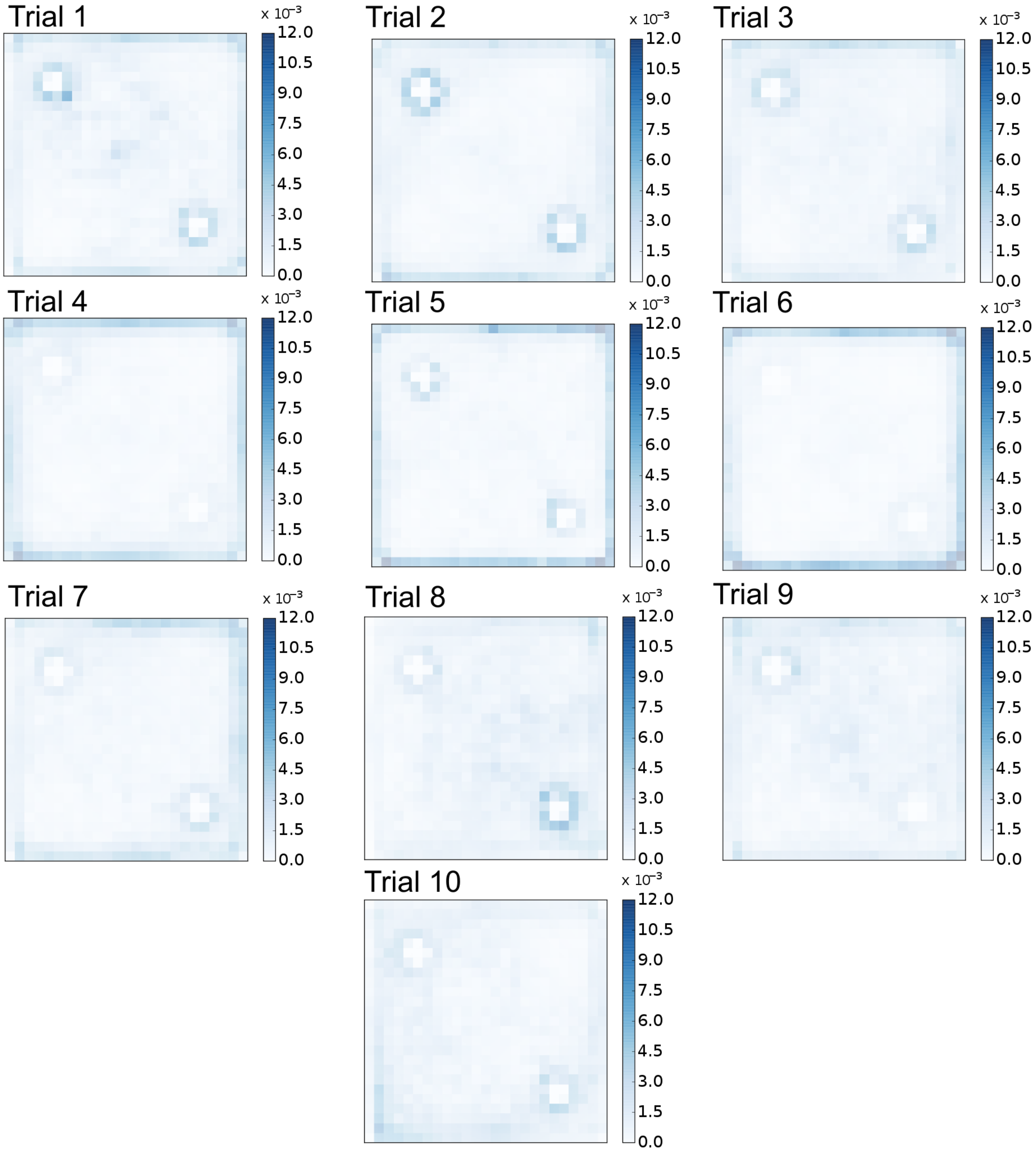

Figure 1: **Probabilities of presence** of 10 trials of 5 AB zebrafish with two cylinders. The probability is calculated on the positions of all zebrafish observed during one hour. The bluer the more fish detected.

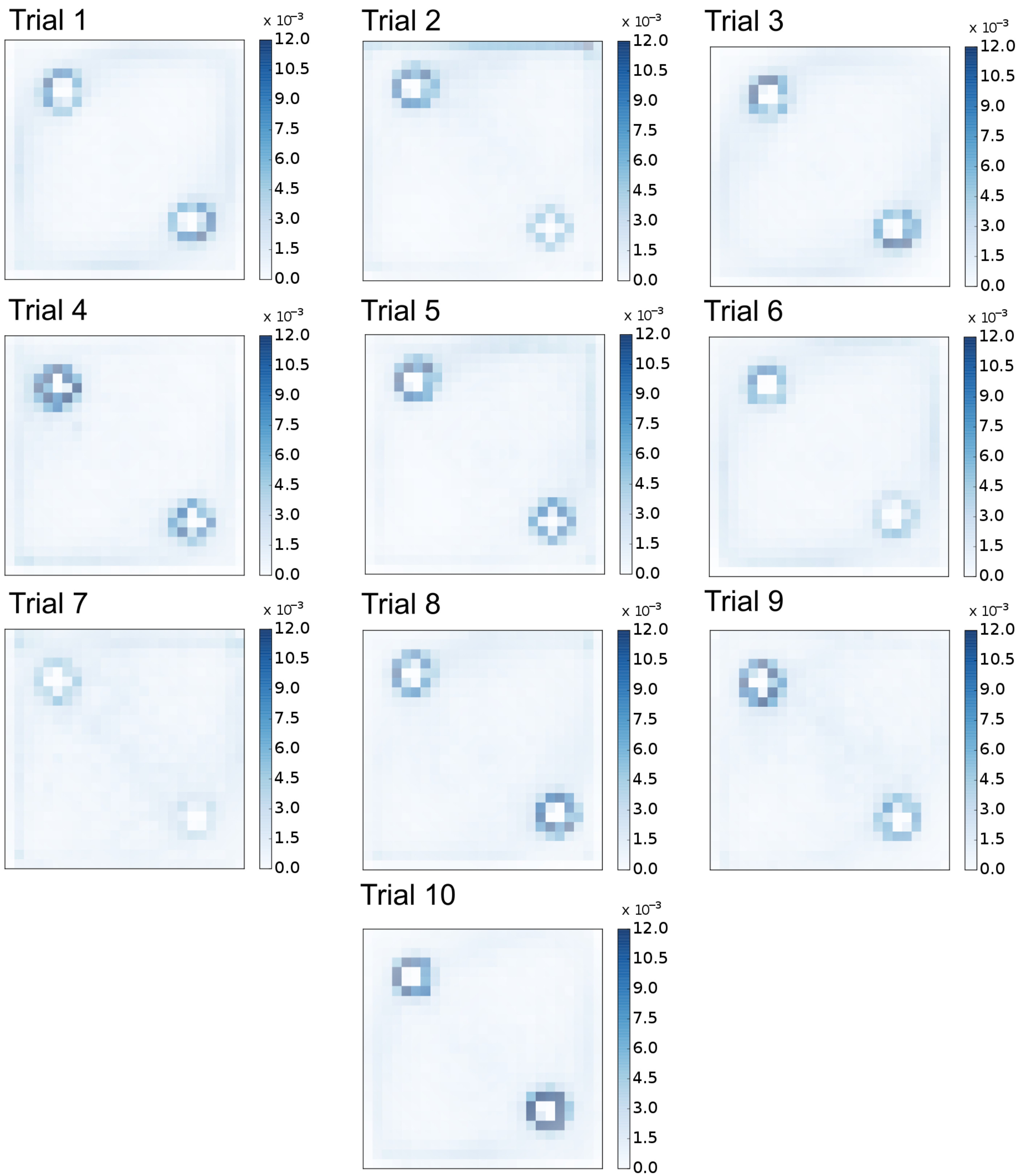

Figure 2: **Probabilities of presence** of 10 trials of 10 AB zebrafish with two cylinders. The probability is calculated on the positions of all zebrafish observed during one hour. The bluer the more fish detected.

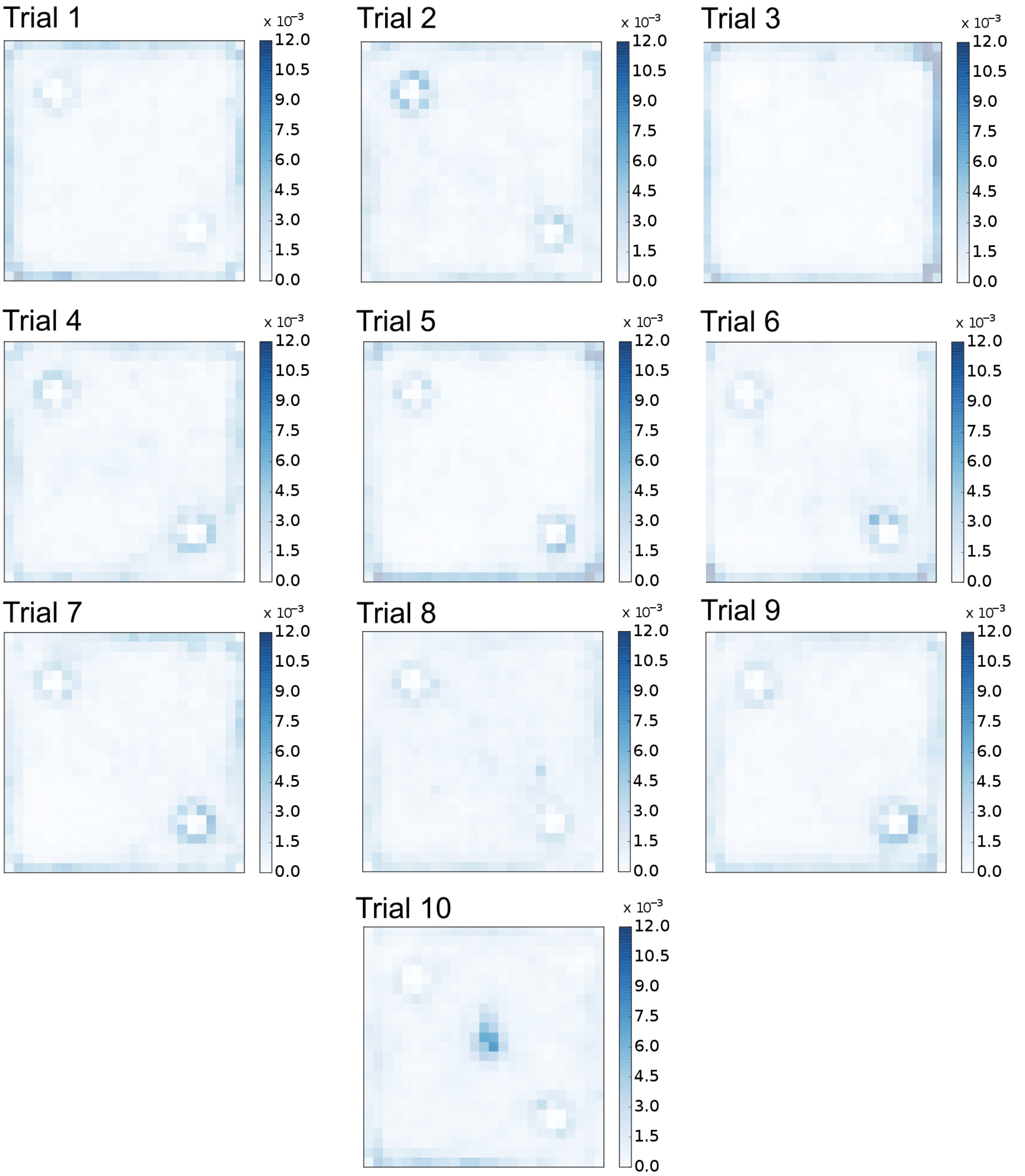

Figure 3: **Probabilities of presence** of 10 trials of 5 TL zebrafish with two cylinders. The probability is calculated on the positions of all zebrafish observed during one hour. The bluer the more fish detected.

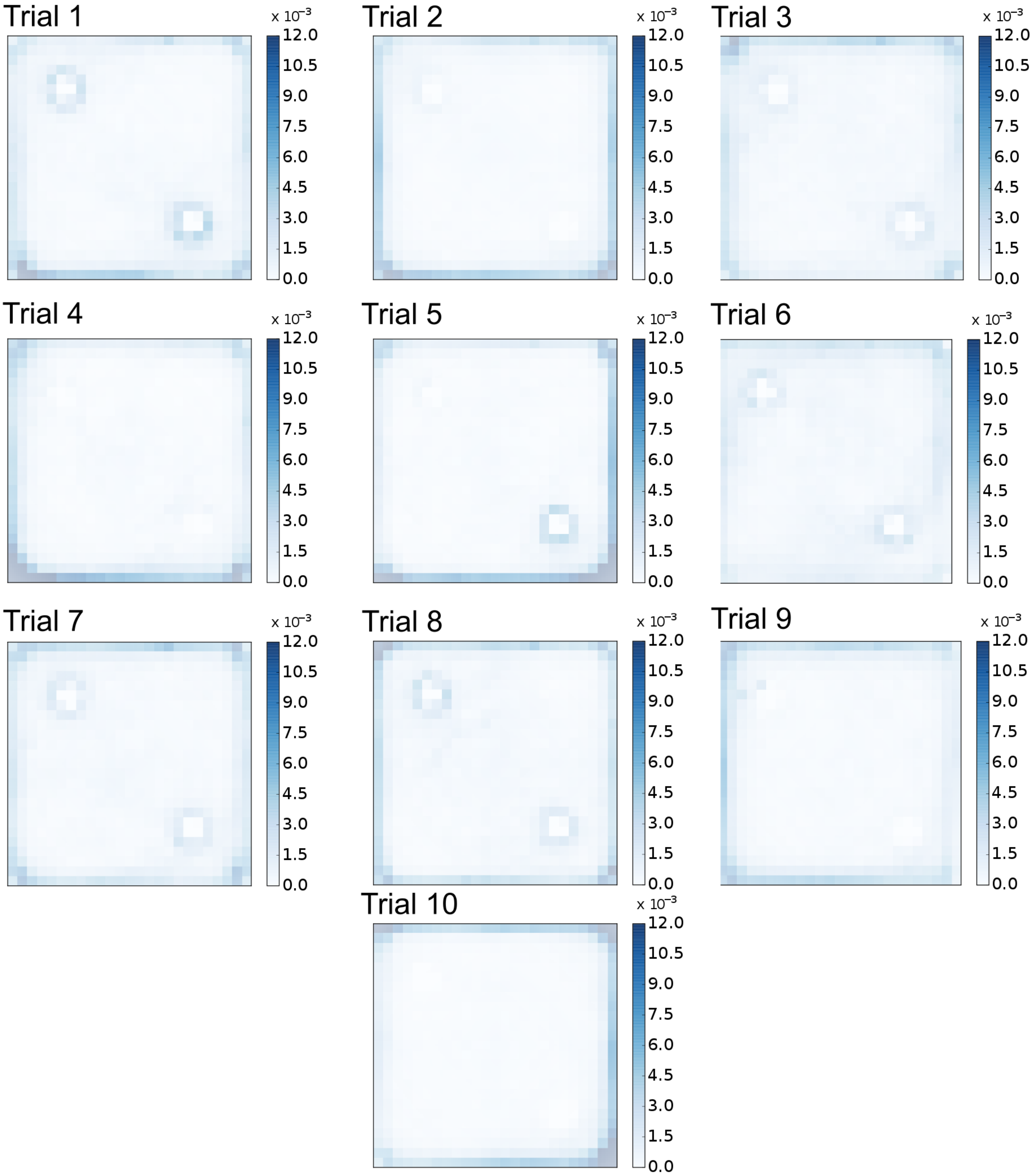

Figure 4: **Probabilities of presence** of 10 trials of 10 TL zebrafish with two cylinders. The probability is calculated on the positions of all zebrafish observed during one hour. The bluer the more fish detected.

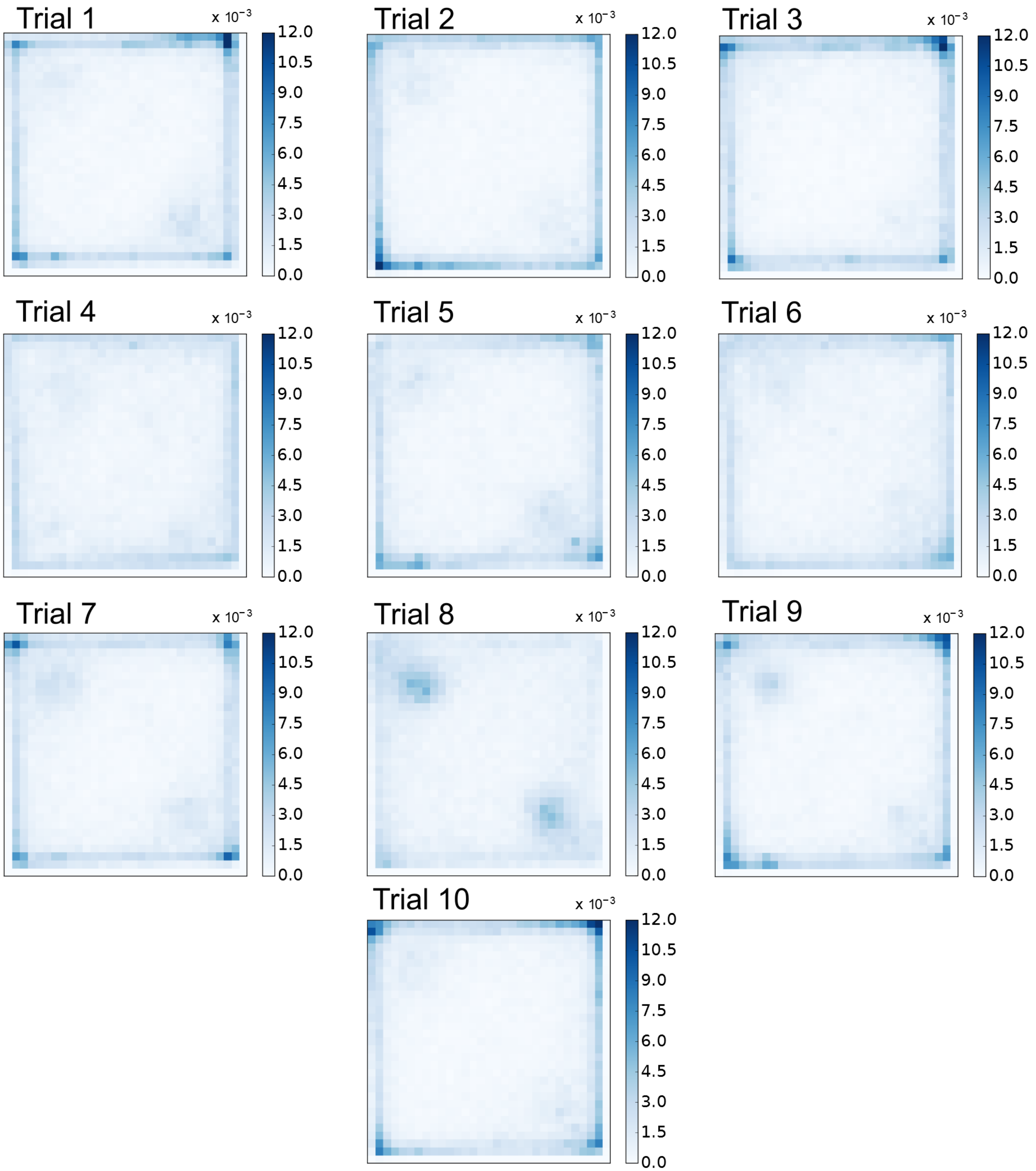

Figure 5: **Probabilities of presence** of 10 trials of 10 TL zebrafish with two disks. The probability is calculated on the positions of all zebrafish observed during one hour. The bluer the more fish detected.

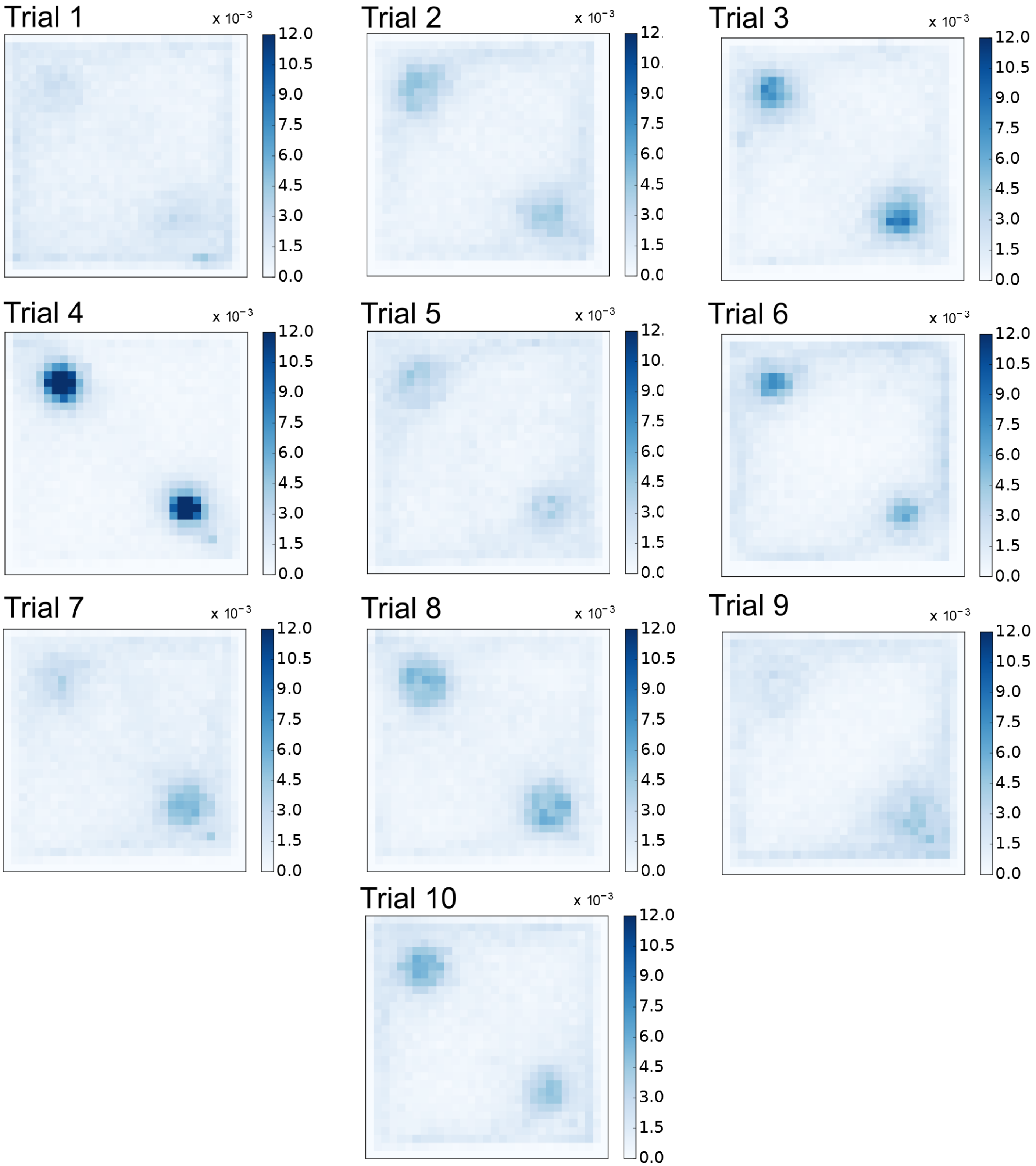

Figure 6: **Probabilities of presence** of 10 trials of 10 AB zebrafish with two disks. The probability is calculated on the positions of all zebrafish observed during one hour. The bluer the more fish detected.

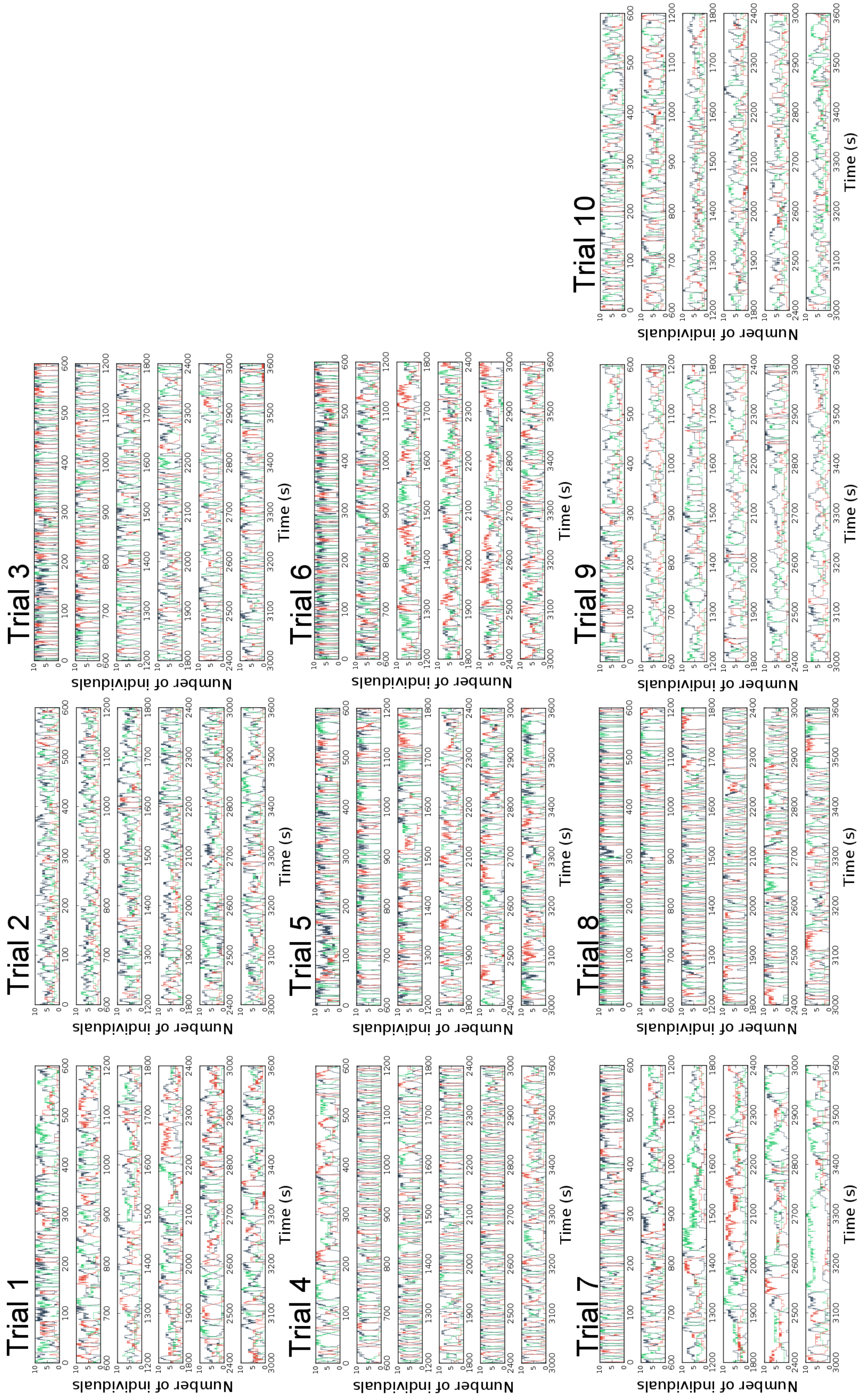

Figure 7: **Landmark occupancy** for 10 trials of 10 AB zebrafish. For readability, time series are divided in 6 linked subplots. Y-axis reports the number of individuals. Green line represents individuals in the zone of interest (less than 25 cm away from the center of the landmark) of landmark 1, red line the individuals in the zone of interest of landmark 2 and blue line the individuals outside both zones of interest.

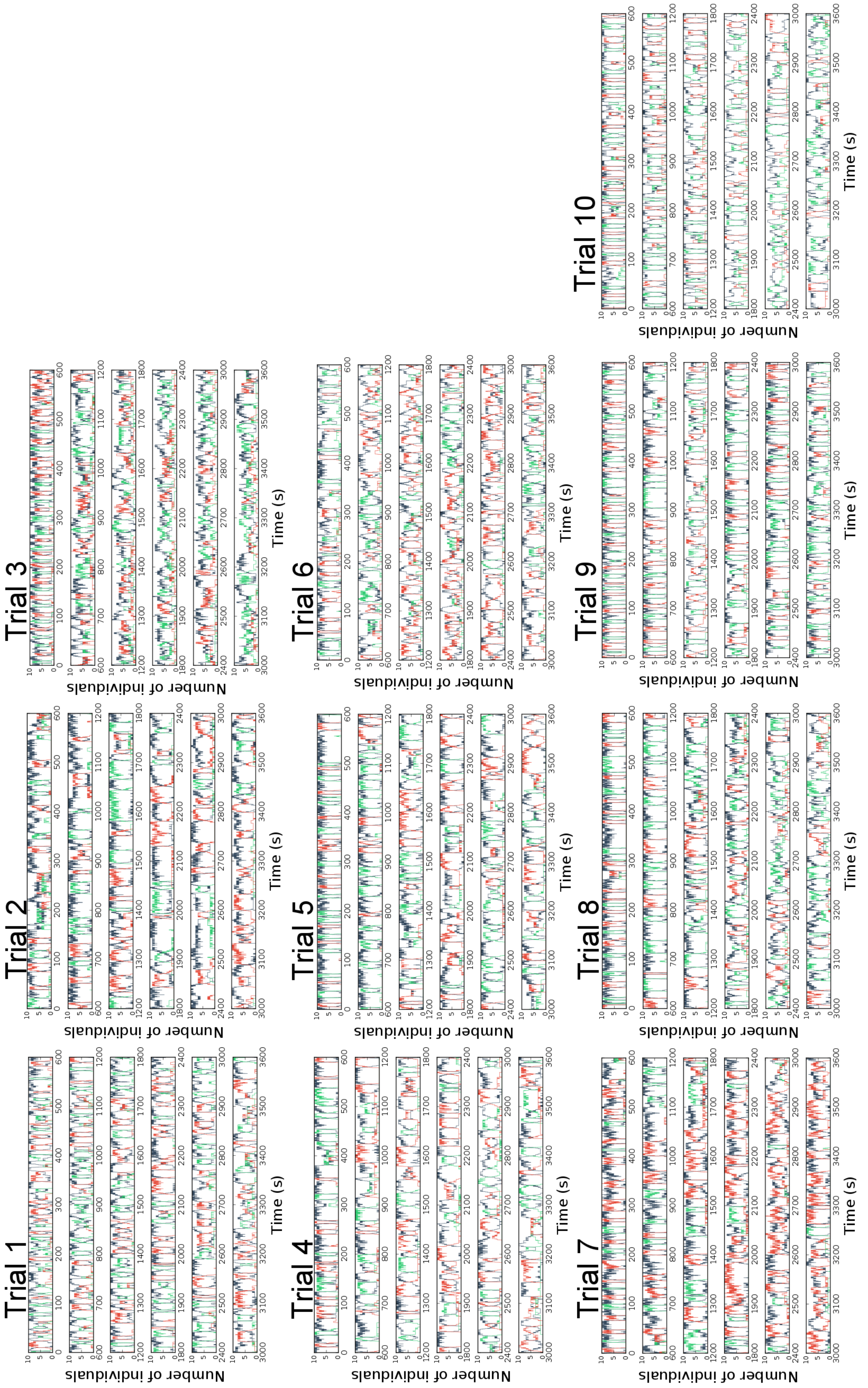

Figure 8: **Landmark occupancy** for 10 trials of 10 TL zebrafish. For readability, time series are divided in 6 linked subplots. Y-axis reports the number of individuals. Green line represents individuals in the zone of interest (less than 25 cm away from the center of the landmark) of landmark 1, red line the individuals in the zone of interest of landmark 2 and blue line the individuals outside both zones of interest.

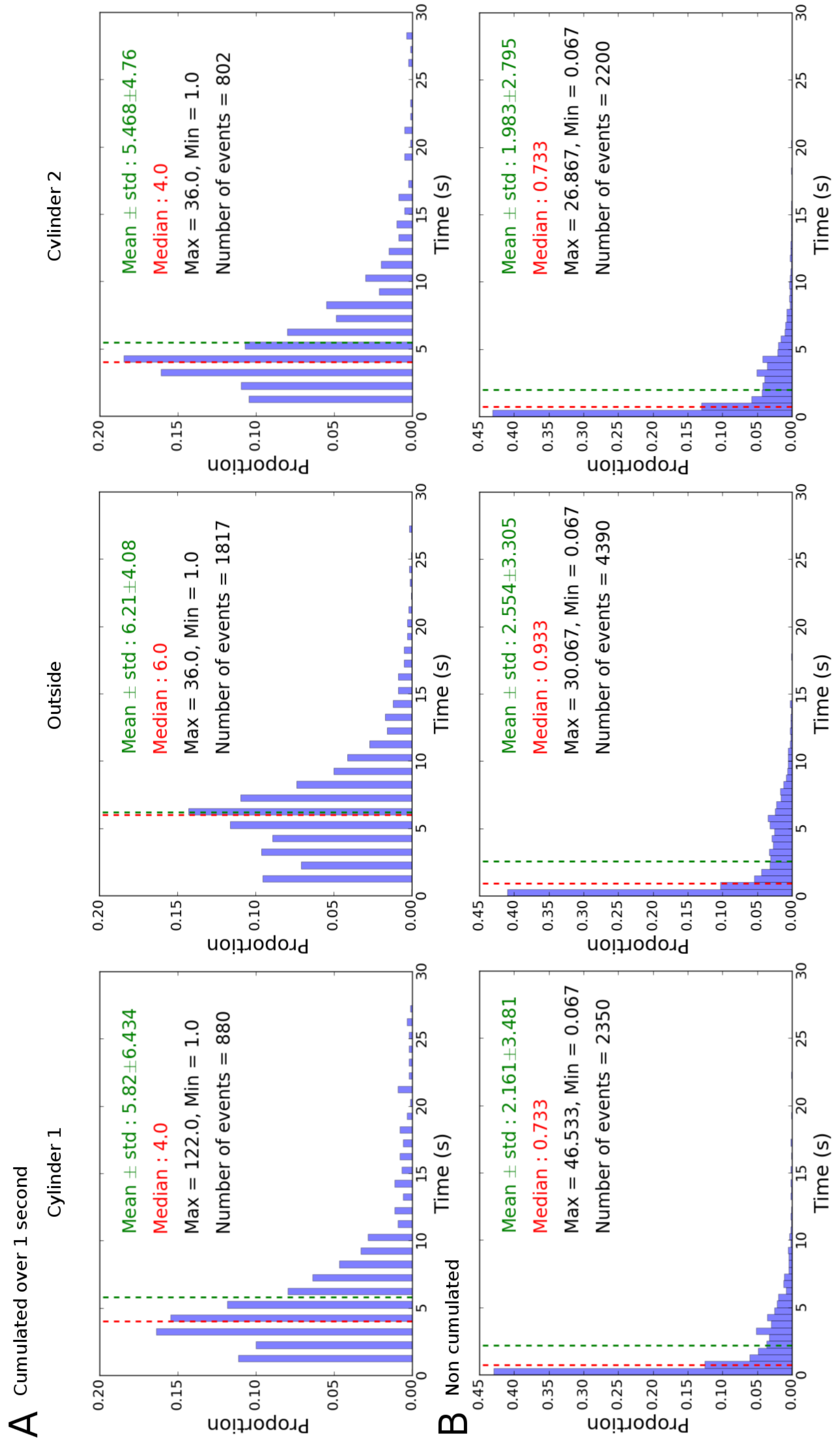

Figure 9: **Comparison of the proportions of the durations of the majority events for AB zebrafish** (A) on cumulated data over 1 second ; (B) on non cumulated data. This figure is related to the Figure 4 of the article. By cumulating the data over 1 second, we decrease strongly the noise. In each area, the median of the presence durations increases and reaches 4 seconds around cylinder 1 and 2 and 6 seconds outside.

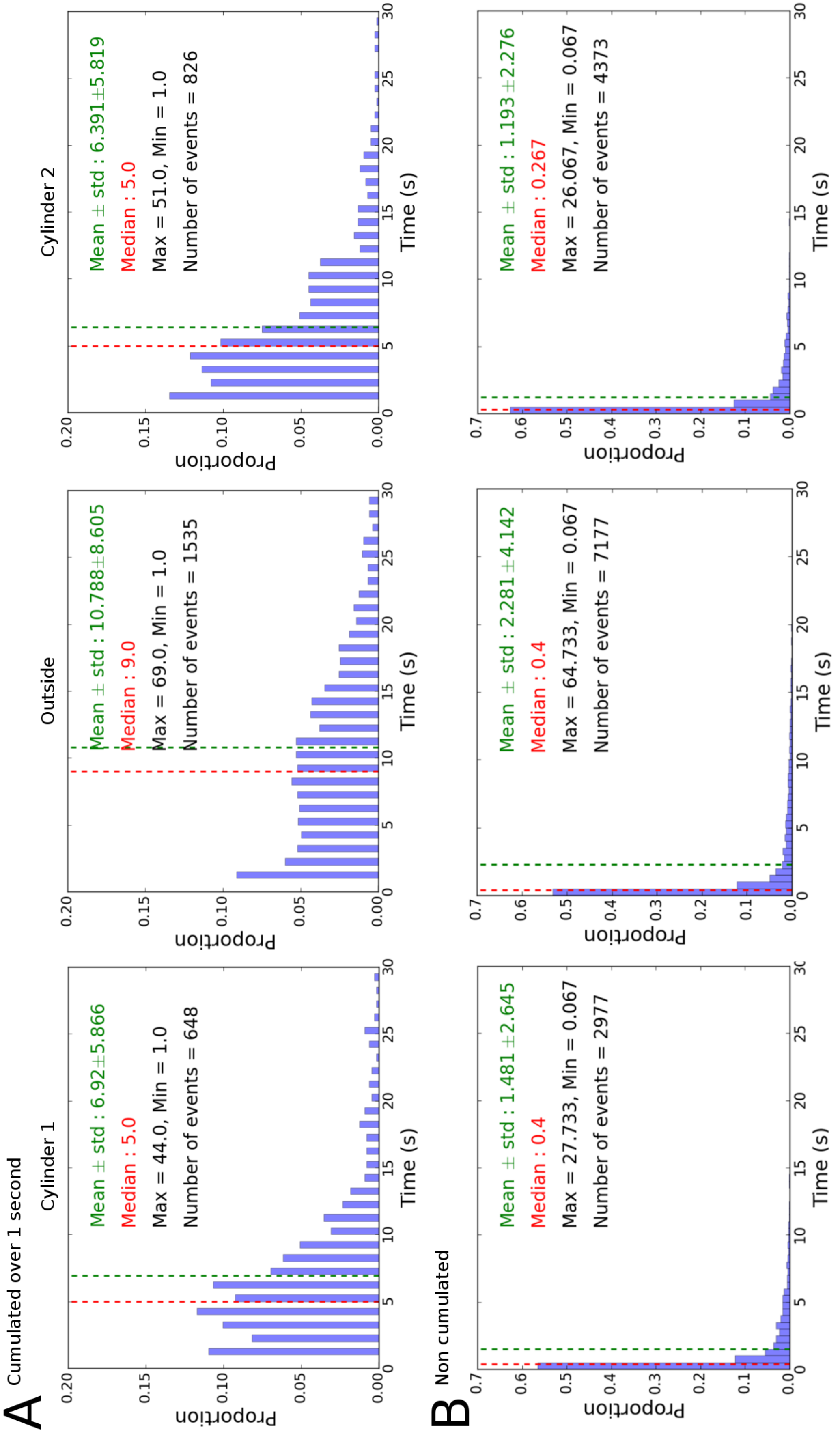

Figure 10: **Comparison of the proportions of the durations of the majority events for TL zebrafish** (A) on cumulated data over 1 second ; (B) on non cumulated data. This figure is related to the Figure 4 of the article. By cumulating the data over 1 second, we decrease strongly the noise. In each area, the median of the presence durations increases and reaches 5 seconds around cylinder 1 and 2 and to 9 seconds outside.

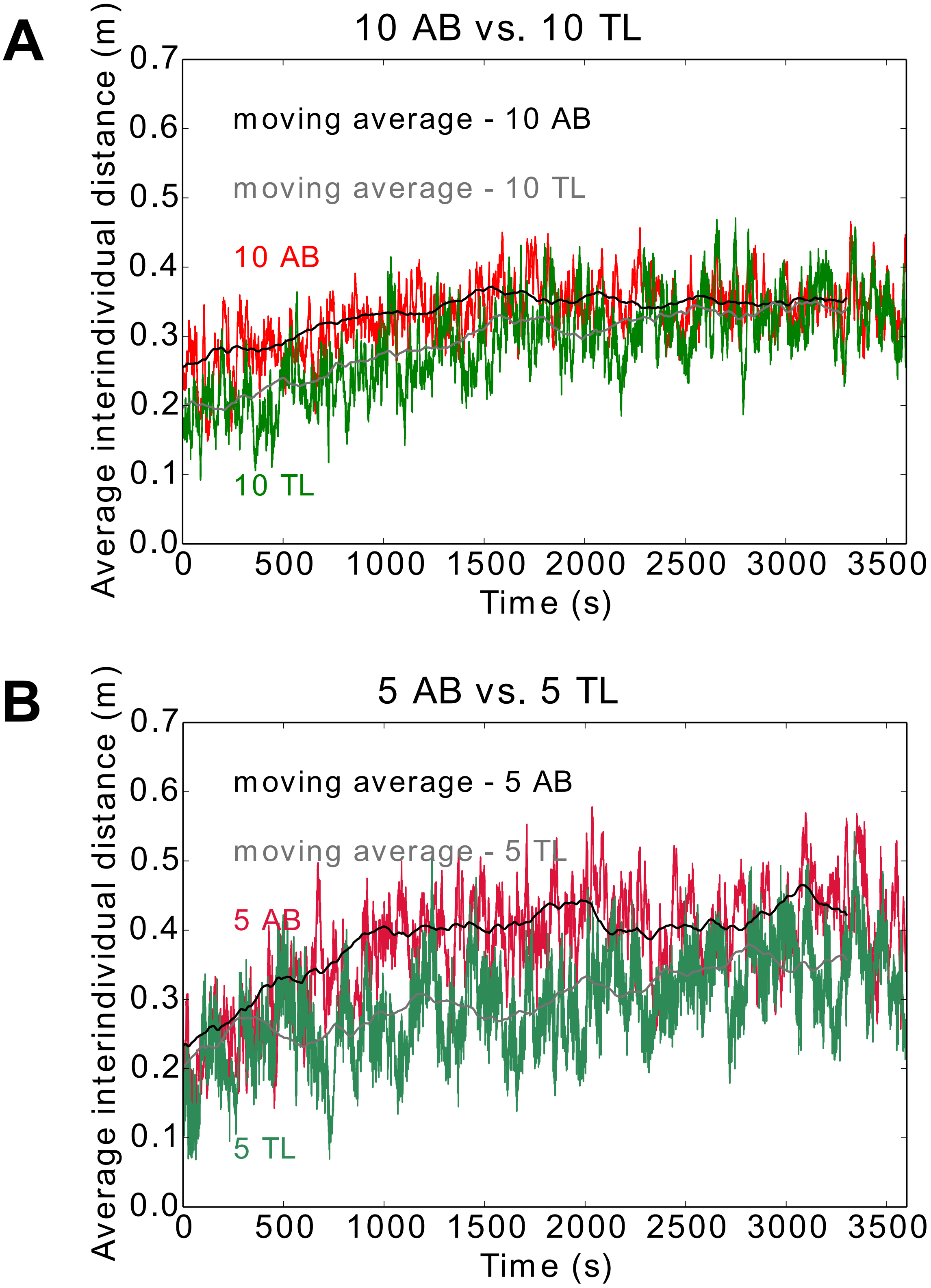

Figure 11: **Time evolution of the average interindividual distance** (A) for 10 AB versus 10 TL fish (in the presence of cylinders). The red line is the average of 10 trials with groups of 10 AB zebrafish, the green line is the average of 10 trials with groups of 10 TL zebrafish ; (B) for 5 AB versus 5 TL fish. The red line is the average of 10 trials with groups of 5 AB zebrafish, the green line is the average of 10 trials with groups of 5 TL zebrafish. The average interindividual distance for each condition shows an increase during the first 20 minutes and the reaching of a plateau. It has been calculated as the average of all distances between each couple of fish.

Experiments	Mean of percentages of tracking efficiencies
10 AB cylinders	96.05%
10 TL cylinders	92.73%
5 AB cylinders	97.80%
5 TL cylinders	91.77%
10 AB disks	100%
10 TL disks	100%

Table 1: **Means of percentages of tracking efficiencies**.

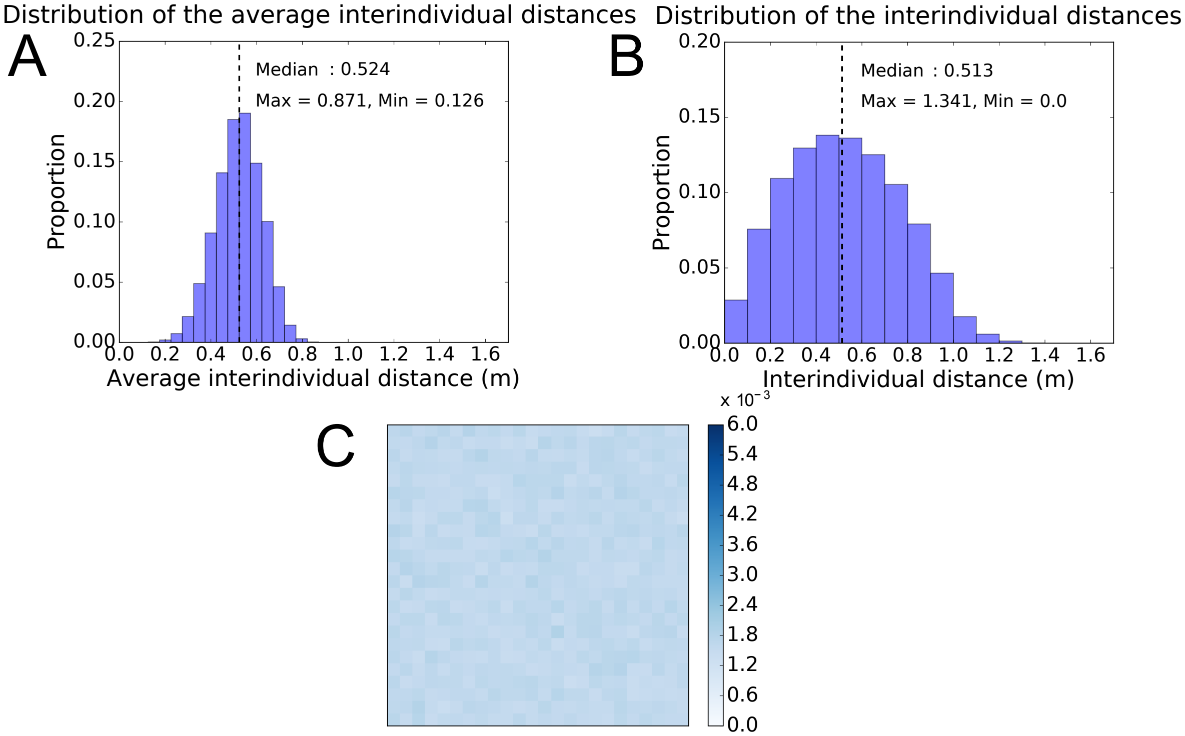

Figure 12: **Randomised data of 5 agents for 10 trials** in a 100 cm x 100 cm arena with two cylinders. (A) The distribution of the average interindividual distances with randomised data can be compared with Fig. 2 (B) of the main article (The medians of the distributions of the average interindividual distances for 5 AB is 0.39m and for 5 TL is 0.30m). (B) The distribution of the interindividual distances with randomised data can be compared with Fig. 1 (C and D) of the main article (The medians of the distributions of the interindividual distances for 5 AB is 0.27m and for 5 TL is 0.12m). (C) The probability of presence of randomised data can be compared with Fig. 5 (C and D) of the main article.

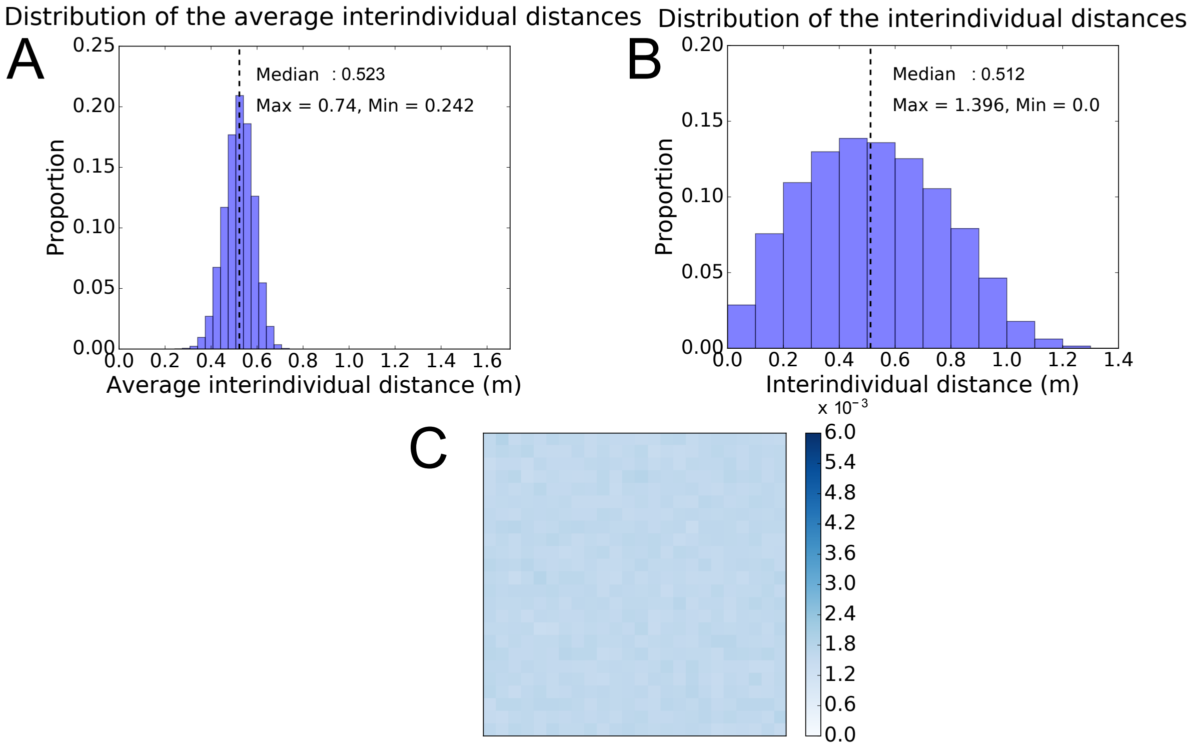

Figure 13: **Randomised data of 10 agents for 10 trials** in a 100 cm x 100 cm arena with two cylinders. (A) The distribution of the average interindividual distances with randomised data can be compared with Fig. 2 (A) of the main article (The medians of the distributions of the average interindividual distances for 10 AB is 0.33m, for 10 TL is 0.29m). (B) The distribution of the interindividual distances with randomised data can be compared with Fig. 1 (A and B) of the main article (The medians of the distributions of the interindividual distances for 10 AB is 0.23m, for 10 TL is 0.14m). (C) The probability of presence of randomised data can be compared with Fig. 5 (A and B) of the main article.

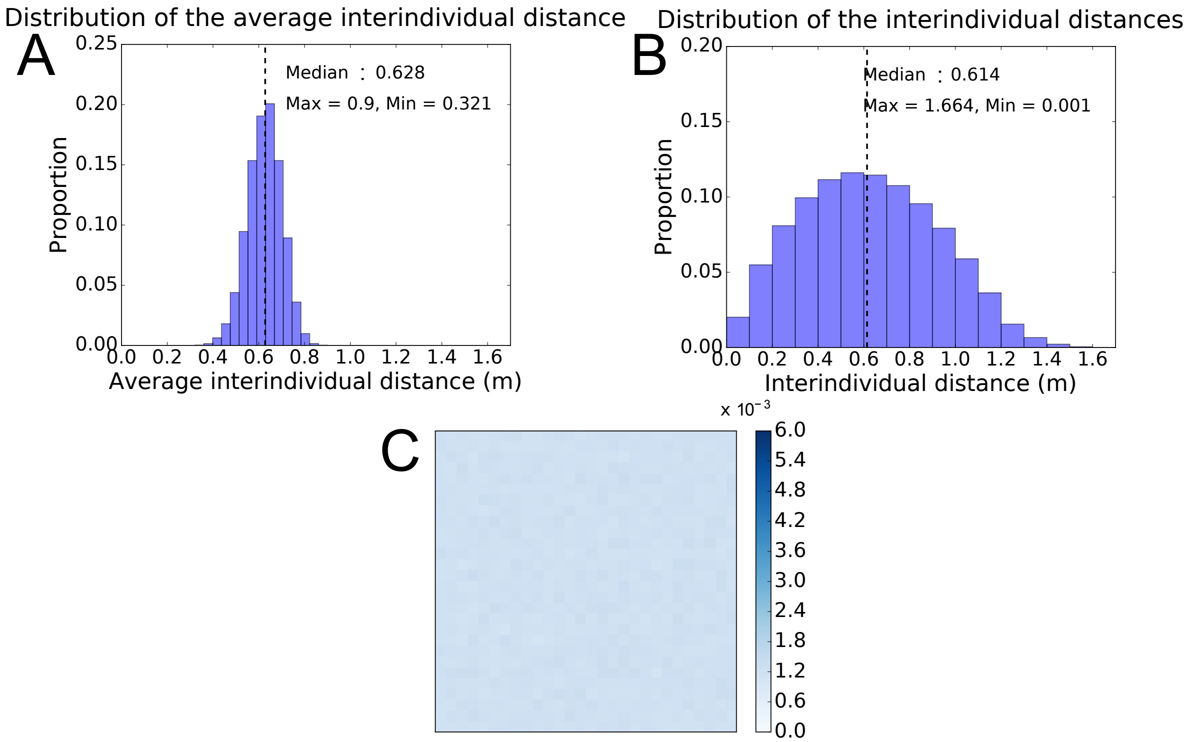

Figure 14: **Randomised data of 10 agents for 10 trials** in a 120 cm x 120 cm arena with two disks. (A) The distribution of the average interindividual distances with randomised data can be compared with Fig. 4 of the main article (The medians of the distributions of the average interindividual distances for 10 AB : 0.45m and for 10 TL : 0.41m). (B) The distribution of the interindividual distances with randomised data can be compared with Fig. 3 (A and B) of the main article (The medians of the distributions of the interindividual distances for 10 AB : 0.35m and for 10 TL : 0.23m). (C) The probability of presence of randomised data can be compared with Fig. 6 (A and B) of the main article.
